# Identification of Metabolites and Metabolic Pathways Related to Treatment with Bufei Yishen Formula in a Rat COPD Model Using HPLC Q-TOF/MS

**DOI:** 10.1155/2015/956750

**Published:** 2015-06-15

**Authors:** Liping Yang, Jiansheng Li, Ya Li, Yange Tian, Suyun Li, Suli Jiang, Ying Wang, Xuekun Song

**Affiliations:** ^1^Henan University of Traditional Chinese Medicine, Zhengzhou 450008, China; ^2^Respiratory Disease Institute, The First Affiliated Hospital of Henan University of TCM, Zhengzhou 450000, China

## Abstract

As a traditional Chinese medicine, Bufei Yishen Formula (BYF) is widely used in China as an effective treatment for chronic obstructive pulmonary disease (COPD). Because of the component complexity and multiple activities of Chinese herbs, the mechanism whereby BYF affects COPD is not yet fully understood. Herein, pulmonary function experiments and histomorphological assessments were used to evaluate the curative effect of BYF, which showed that BYF had an effect on COPD. Additionally, a high performance liquid chromatography quadrupole time-of-flight mass spectrometry (HPLC QTOF/MS) metabonomics method was used to analyze the mechanism of the actions of BYF on rats with COPD induced by a combination of bacteria and smoking. Partial least squares discriminate analysis (PLS-DA) was used to screen biomarkers related to BYF treatment. Candidate biomarkers were selected and pathways analysis of these metabolites showed that three types of metabolic pathways (unsaturated fatty acid metabolism-related pathways, phenylalanine metabolism-related pathways, and phospholipid metabolism-related pathways) were associated with BYF treatment. Importantly, arachidonic acid and related metabolic pathways might be useful targets for novel COPD therapies.

## 1. Introduction

Chronic obstructive pulmonary disease (COPD) is a slowly progressive and poorly reversible disease that is characterized by an abnormal inflammatory response in the lung [1]. Cigarette smoking, air pollutants, dust, and inherent susceptibility are all risk factors for COPD development [1]. Notably, air pollution of Chinese cities is becoming increasingly serious as the Chinese economy has grown, resulting in a significantly increased prevalence of COPD in China [2]. It has been reported that COPD affects 8% of Chinese individuals older than 40 [3], and it is predicted to become the third most frequent cause of death worldwide by 2020 [4]. In Western medicine, some glucocorticoids and bronchodilators can alleviate the acute onset of COPD, but significant side effects exist [5–8]. Therefore, the development of novel longer lasting, targeted therapeutic strategies is urgently needed.

In China, traditional herbal formulas have been used for many centuries to treat COPD, and clinical trials and experimental studies have shown that certain Chinese medicines can effectively treat COPD by improving pulmonary function, respiratory muscle fatigue, immunity, and lung blood flow [9–11]. Moreover, compared to Western medicine, many Chinese herbs have few side effects [12]. We previously showed that a traditional Chinese medicine (TCM), Bufei Yishen Formula (BYF), had a curative effect on COPD, and the molecular mechanisms of its effect on COPD were investigated using transcriptomics (microarray analysis). We found that BYF treatment could significantly improve pulmonary function and lung tissue pathology of COPD rats by reducing the expression level of inflammation-related interleukins [13].

Herein, we explored the curative effect and molecular mechanism of BYF treatment using a new systems biology platform, metabonomics. This technology can make qualitative and/or quantitative measurements of metabolites in living systems and simultaneously detect many metabolites related to physiological or exogenous stimulations. Such an approach is predicted to accelerate pharmacological research, such as drug target screening and validation, mechanistic research, and medicinal evaluations [14–17]. A modified COPD rat model was established, as described in our previous study [13] and by Chen et al. [18], and three experimental groups (COPD, BYF, and controls) were used to compare pulmonary function and pathology. High performance liquid chromatography/quadrupole time-of-flight mass spectrometry (HPLC QTOF/MS) was used to identify differential metabolites (DMs) between the three experimental groups. Additional bioinformatics analyses were used to study the relationships between these DMs and BYF treatment.

## 2. Materials and Methods

### 2.1. BYF Preparation and Animal Model Establishment

BYF was prepared at the First Affiliated Hospital of Henan University of Traditional Chinese Medicine (Zhengzhou, Henan, China). BYF included 15 g Huangqi (*Radix Astragali*), 15 g Renshen (*Radix Ginseng*), 15 g Shanzhuyu (*Fructus Corni*), and 9 g Wuweizi (*Fructus Schisandrae*). All herbs were decocted with water, steam sterilized, and brought to a final concentration of 0.6 g/mL. BYF has been described in a previous study [13] and a Chinese patent (number 2011101175781).

Experimental protocols were approved by the Experimental Animal Care and Ethics Committees of the First Affiliated Hospital, Henan University of Traditional Chinese Medicine. Two-month-old Sprague-Dawley rats that weighed 200 ± 20 g were purchased from Henan Experimental Animal Center (Henan XK2005-0001). They were maintained on a 12 h dark/light cycle with an ambient temperature of 25 ± 1°C, a relative humidity of 50 ± 10%, and sufficient food (sterile rat chow) and water (sterile). All rats were anesthetized and sacrificed under the experimental protocols mentioned above and all efforts were made to minimize suffering.

**Figure 1:**
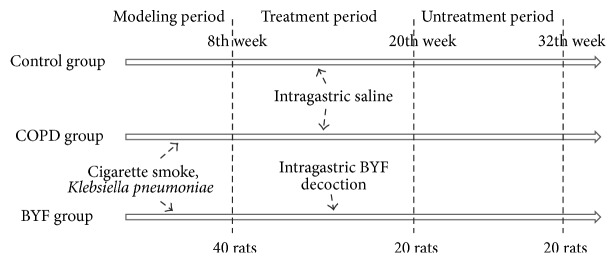
Establishment of an animal model of COPD.

A total of 120 Sprague-Dawley rats were randomized and divided into three experimental groups (control, COPD, and BYF) with equal numbers of males and females in each group. As shown Figure 1, the experimental process can be divided into three periods. (1) In the modeling period from weeks 0 to 8, in which 40 rats in the control groups were not treated, all rats in the COPD and BYF groups underwent intranasal instillation with* Klebsiella pneumoniae* once every 5 days for 8 weeks. Rats were placed in a 300 L smoke box for 30 min at 3 h intervals between smoke treatments, wherein eight cigarettes were burned twice daily in the first two weeks, and 15 cigarettes were burned three times daily during weeks 3 to 8. (2) In the treatment period, from weeks 9 to 20, all rats in the control and COPD groups were administered 2 mL intragastric saline vehicle (0.9%) twice daily, but all rats in the BYF group received 4.44 g/kg/d BYF twice daily. At the end of week 20, half of the rats (20) in each of the three groups were selected and sacrificed for subsequent experiments. (3) In the untreatment period, from weeks 21 to 32, all other rats in the three groups were raised to week 32 without additional treatment. All rats were weighed weekly to determine dosing during the 32-week period.

### 2.2. Preparation and Determination of Pulmonary Function and Pathology

Tidal volume (TV), peak expiratory flow (PEF), and 50% tidal volume expiratory flow (EF50) were determined by unrestrained pulmonary function testing plethysmographs (Buxco Inc., Wilmington, NC, USA) conducted at 4-week intervals from weeks 0 to 32. Paraffin-embedded sections of lung tissue were stained with hematoxylin and eosin and images were obtained by light microscopy (Olympus, Tokyo, Japan).

### 2.3. Sample Preparation

At weeks 20 and 32, eight lung tissue samples were excised from each of the three experimental groups for metabonomics analysis. Samples of 60 ± 5 mg lung tissue were collected from each rat. To each sample, 200 *μ*L methanol (–20°C) was added and tissues were homogenized with a Qiagen Tissuelyzer II (30 Hz, 3 min) and then mixed by shaking with 200 *μ*L chloroform, 300 *μ*L methanol, and 200 *μ*L double-distilled water. Then, samples were centrifuged (4°C, 14,000 rpm, 15 min) to separate tissue debris, mixed by shaking the tissue debris with 500 *μ*L methanol and then centrifuged again (4°C, 14,000 rpm, 15 min) to remove tissue debris. Next, supernatants from the two centrifugation steps were combined and volatilized in 500 *μ*L supernatant with moderate nitrogen and then added to a 100 *μ*L methanol : water (1 : 1) mixture and centrifuged (4°C, 14,000 rpm, 15 min). The resulting supernatant was injected into a HPLC Q-TOF/MS system for analysis.

### 2.4. HPLC Q-TOF/MS Analysis

An Agilent HPLC 1200 system equipped with a binary solvent delivery manager and a sample manager (Agilent Corp., Santa Clara, CA, USA) was used with chromatographic separations performed on a 2.1 × 50 mm 2.7 *μ*m Poroshell SB-C18 reversed phase column. The LC elution conditions were optimized as follows: isocratic at 1% B (0–3 min), 1–45% B (3–9 min), 45–80% B (9–11 min), and 80–100% B (11–18 min), isocratic at 100% B (18-19 min); linear gradient from 100% to 1% B (19-20 min) and isocratic at 1% B (20–25 min). Here, A = water with 0.1% formic acid and B = acetonitrile with 0.1% formic acid. The column was maintained at 40°C. A 5 L aliquot sample was then injected onto the column.

Mass spectrometry was performed using an Agilent model 6520 QTOF mass spectrometer equipped with a dual sprayer electrospray ionization source (Agilent). The experiments were carried out in the positive ESI mode using the following operating parameters: drying gas (N_2_) flow rate, 10 L/min; drying gas temperature, 330°C; nebulizer, 40 psi; capillary, 4000 V; and skimmer, 65 V. Data acquisition was performed using Agilent Mass Hunter Acquisition Software. Mass spectra were recorded across a range of 100–1000 Da with accurate mass measurements of all mass peaks.

### 2.5. Data Preprocessing

The resulting files were centroided, deisotoped, and converted into mzData xml files using the MassHunter Qualitative Analysis Program (vB.03.01; Agilent). Following conversion, xml files were analyzed using the open source XCMS package (v1.24.1; http://metlin.scripps.edu/), which runs in the statistical package R (v.2.12.1; http://www.r-project.org/), to select, align, and quantify features (chromatographic events corresponding to specific* m/z* values and retention times). The resulting 3D matrix, containing an arbitrarily assigned peak index, the retention time, and abundance values (.xls file), were then exported to SIMCAP software 12.0 (Umetrics, Umeå, Sweden) for multivariate statistical analysis. Mean-centered and par-scaled (scaled to the square root of SD) mathematical methods were performed to pretreat datasets. Partial Least Squares Discriminant Analysis (PLS-DA) was used to visualize general clustering, trends, and outliers among the observations.

Differential metabolites (DMs) between groups were screened based on a threshold of variable importance in the projection (VIP) value (VIP > 1) based on the PLS-DA model. In parallel, these DMs from the PLS-DA model were validated at a univariate level using Student's *t*-test (*P* < 0.05). Compound identification was performed using METLIN (http://metlin.scripps.edu/). Analyses of metabolite pathways were based on the Kyoto Encyclopedia of Genes and Genomes (KEGG) using MetPA software [19].

## 3. Results and Discussion

### 3.1. Pulmonary Function and Pathology Improvements in COPD Rats Treated with BYF

From 0 to 32 weeks, control group rats were kept active and restless and exhibited smooth and burnished fur. The body mass of control group rats increased gradually and respiration remained stable. By contrast, in the modeling period (0 to 8 weeks), rats in the COPD and BYF groups exhibited signs of malaise, which were characterized by appetite suppression and wriggling with gathered fur. The body mass of these rats slowly increased with short respiration that was accompanied by frequent coughs. These symptoms in the COPD group lasted until the end of the 32-week study period, but these symptoms in the BYF group rats were significantly alleviated following BYF treatment in both the treatment (9 to 20 week) and untreated periods, in which BYF treatments were stopped from 20 to 32 weeks.

**Figure 2:**
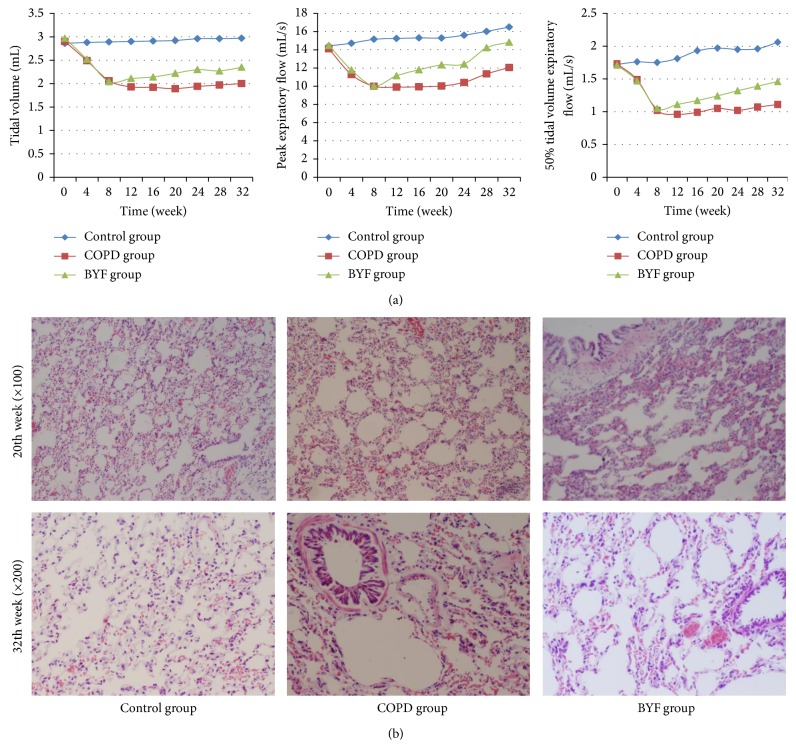
Pulmonary function and pathology in each experimental rat group. (a) The results of TV, PEF, and EF50 for the three groups during the 32-week study period. (b) Lung histomorphological observations by light microscopy for the three groups at weeks 20 and 32.

Pulmonary function for all three experimental groups was detected via TV, PEF, and EF50 at 4-week intervals for 32 weeks. TV, PEF, and EF50 were found to be stable in the control group but were dramatically reduced during the modeling period (0 to 8 weeks) in the COPD and BYF groups (*P* < 0.05). Following BYF treatment, TV, PEF, and EF50 in the BYF group were significantly improved (all *P* < 0.05; Table 1; Figure 2(a)) compared to rats in the COPD group, and these parameters showed continuous improvement during the untreatment period (20 to 32 weeks, when BYF treatment was stopped).

Histological analyses of lung tissues harvested from each of the three experimental groups at weeks 20 and 32 week are shown in Figure 2(b). Compared to controls, COPD rats at week 20 showed upregulation of a severe inflammatory response with visible increases in lymphocytes, monocytes, and neutrophils. Bronchial and pulmonary wall thickness, the degree of bronchial stenosis, and the alveolar diameter were significantly greater in the COPD group, while the alveolar count per unit area was significantly lower compared to control rats; these symptoms all worsened by week 32. Notably, these COPD-related phenomena were markedly relieved by BYF treatment by weeks 20 and 32. Furthermore, BYF treatment significantly alleviated the inflammatory response, as shown by a significant reduction in the number of inflammatory cells that were present in lung tissues.

Based on visual inspections, pulmonary function experiments, and lung histological analyses, we found that BYF treatment could substantially improve various symptoms of COPD. Furthermore, these COPD-related symptoms continued to resolve, even after treatment was halted during the untreatment period, indicating that BYF exerted a long lasting effect on COPD rats.

### 3.2. Multivariate Analysis of the Metabonomics Data

**Figure 3:**
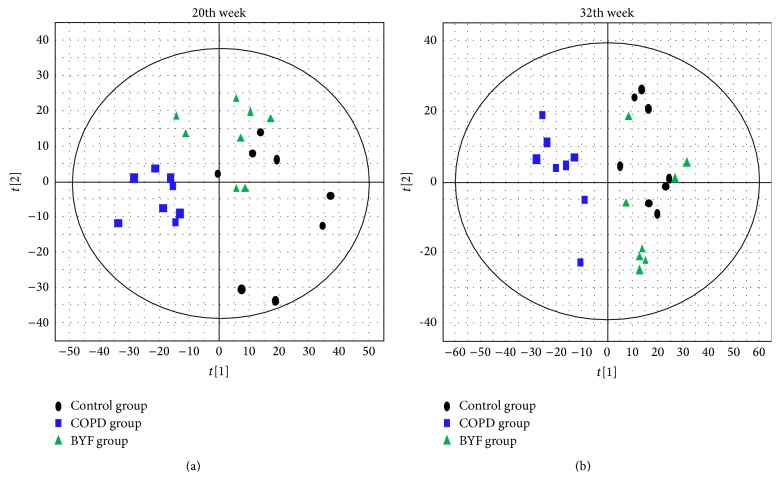
PLS-DA score plots obtained from the three groups at weeks 20 and 32. For week 20: R2X = 0.785, R2Y = 0.53, and Q2 = 0.389; for week 32: R2X = 0.618, R2Y = 0.517, and Q2 = 0.536.

Lung tissues from the three groups (eight rats per group) were collected for the detection of metabolites by HPLC Q-TOF/MS. We selected two time points (weeks 20 and 32) for these experiments. PLS-DA was used for multivariate data analysis.  Figure 3 shows the PLS-DA score plot among the control, COPD, and BYF groups at weeks 20 and 32. We observed that at week 20, the plots for the COPD group were inclined to be tightly and independently clustered, whereas the control and BYF group plots were relatively dispersed and overlapping. Nevertheless, at week 32, we observed that the COPD group plots remained clustered and independent, but the control and BYF group plots showed more overlap. This finding demonstrated that the metabolites in the COPD rats were perturbed by the COPD pathological state (control/COPD) and the BYF treatment rats, which gradually returned to normal levels based on the plots of the control and BYF groups, remained similar to each other (week 32/week 20). Furthermore, our findings were in accordance with the variation observed in the pulmonary function experiments and pathological images. We speculate that the changes in COPD symptoms could be highly related to changes in the levels of endogenous metabolites. Accordingly, BYF treatment had a substantial effect on the COPD rats.

### 3.3. Biomarkers Related to BYF Treatment

To characterize the molecular mechanism of BYF, the DMs between the three groups were identified using PLS-DA (VIP > 1, *t*-test *P* < 0.05). At the week 20 time point, 49 and 46 DMs were detected between the control and COPD groups and the COPD and BYF groups (Table S1; see Supplementary Material available online at http://dx.doi.org/10.1155/2015/956750), respectively. At the week 32 time point, 40 and 32 DMs were detected between the control and COPD groups, and the COPD and BYF groups (Table S1), respectively. To search for candidate biomarkers related to BYF treatment, those DMs that showed opposite changing trends between the COPD/control and BYF/COPD groups were selected. We identified 14 and 5 metabolites as potential biomarkers for the BYF treatment at the two time points (weeks 20 and 32; Table 2), respectively. Intriguingly, arachidonic acid was a common biomarker at both time points, and it was distinctly elevated in the COPD group and reduced in the BYF group at both time points, indicating that arachidonic acid might be a key compound for BYF treatment. To date, many studies have found that the arachidonic acid and corresponding derivatives are related to COPD [20, 21]. Arachidonic acid and corresponding derivatives have been considered to act as an “inflammatory switch” in COPD [22]. Moreover, Drakatos et al. suggested that leukotrienes (derivatives of arachidonic acid) could be a therapeutic target in COPD [23]. In this present study, we found that BYF could distinctly reduce the content of arachidonic acid in the lung tissues of COPD rats, indicating that arachidonic acid might be the molecular target of BYF.

### 3.4. Pathway Analysis of Biomarkers

**Figure 4:**
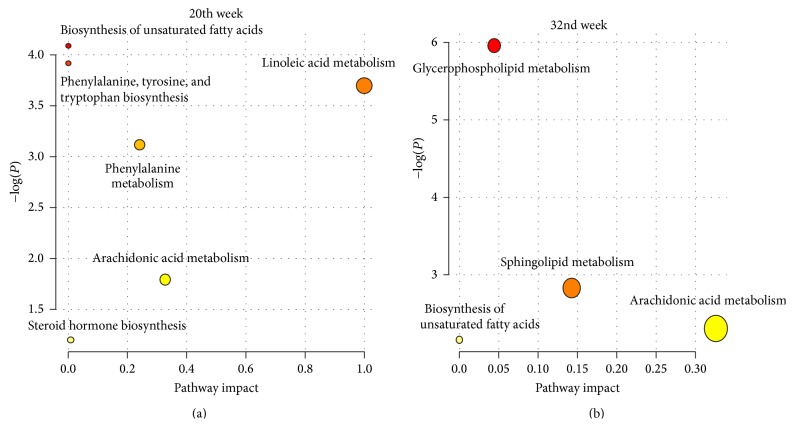
Pathway enrichment analysis of biomarkers at two time points using MetPA.

To facilitate understanding the biological implications of the biomarkers, pathway analyses were performed using MetPA [19], a web-based tool for the analysis and visualization of metabolomic data within the context of metabolic pathways. At week 20, the 14 selected biomarkers were enriched in unsaturated fatty acid metabolism-related pathways (biosynthesis of unsaturated fatty acids, linoleic acid metabolism, arachidonic acid metabolism, and steroid hormone biosynthesis) and phenylalanine metabolism-related pathways (phenylalanine, tyrosine, and tryptophan biosynthesis, phenylalanine metabolism; Figure 4, Table 3). At week 32, the 5 selected biomarkers were also enriched in unsaturated fatty acid metabolism-related pathways (arachidonic acid metabolism and biosynthesis of unsaturated fatty acids; Figure 4, Table 3) and in phospholipid metabolism (glycerophospholipid metabolism and sphingolipid metabolism). Because COPD is accompanied by pulmonary inflammation and phospholipid decline [24, 25], BYF treatment might improve these COPD symptoms by regulating the functions mentioned above.

For unsaturated fatty acid metabolism-related pathways, many studies have established that COPD has a high correlation with arachidonic acid metabolism, linoleic acid metabolism, and steroid hormone biosynthesis. Balode et al. proposed that arachidonic acid metabolism represents a key switch in the inflammation associated with COPD [22], and Drakatos et al. suggested that leukotrienes (derivatives of arachidonic acid) could be therapeutic targets for COPD [23]. Some derivatives of arachidonic acid, including leukotrienes (LTC_4_, LTD_4_, and LTE_4_), are known to induce mucus secretion, cause inflammatory cell infiltration, increase vascular permeability and tissue edema, damage ciliary, and cause severe bronchoconstriction [23]. Additionally, some studies found that linoleic acid plays important anti-inflammatory roles and could also improve respiration in COPD patients [26, 27]. Moreover, some types of glucocorticoids have been used to treat COPD patients and were generally different types of steroid hormones [28–30]. For phenylalanine metabolism-related pathways, it has been reported that the phenylalanine content was lower in COPD patients [31]. Other studies have examined the relationship between phenylalanine receptors and COPD [32]. For phospholipid metabolism-related pathways, Moré et al. found that the phospholipid content was significantly lower in bronchoalveolar fluids from smokers and COPD patients [25]. Thimmulappa et al. reported that abnormal phospholipid metabolism could result in poor pulmonary antibacterial innate immune defenses [33].

As indicated by our previously mentioned findings, BYF treatment could markedly improve inflammation and respiration in COPD by targeting unsaturated fatty acid metabolism-related molecules and pathways, especially arachidonic acid-related metabolism. Furthermore, BYF could also enhance pulmonary antibacterial capacity by affecting both phenylalanine and phospholipid metabolism.

## 4. Conclusion

**Figure 5:**
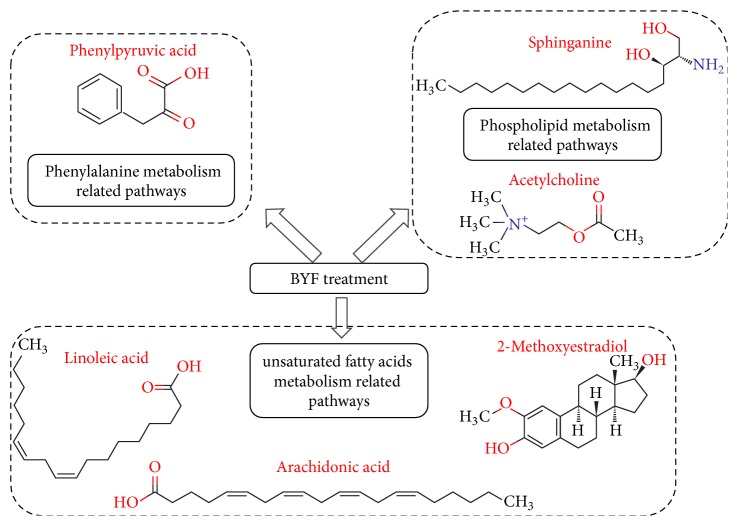
The major pathways and corresponding metabolites disturbed by BYF treatment.

In our present study, three experimental groups were established to investigate the curative effect of BYF in COPD rats. We used pulmonary function experiments and pathological imaging to show that BYF had significant effects on COPD rats, including a long-term effect that persisted after stopping treatment. Meanwhile, to investigate the molecular mechanism whereby BYF could affect COPD rats, a metabonomics approach using HPLC Q-TOF/MS was established. We selected 14 and 5 metabolites as biomarkers that were related to BYF treatment at two different time points, weeks 20 and 32, respectively. Among these compounds, arachidonic acid was found to be related to two time points and could represent the key molecular target of BYF. Pathway analysis using MetPA showed that BYF treatment was performed by correcting three main types of metabolic processes (unsaturated fatty acid metabolism-related pathways, phenylalanine metabolism-related pathways, and phospholipid metabolism-related pathways) and related key metabolites, including linoleic acid, arachidonic acid, 2-methoxyestradiol, phenylpyruvic acid, sphinganine, and acetylcholine (Figure 5).

## Supplementary Material

Table S1. The DMs among three rat groups. sheet 1:the DMs between control and COPD groups in 20th week, sheet 2: the DMs between control and COPD groups in 32th week, sheet 3: the DMs between COPD and BYF groups in 20th week, sheet 4: the DMs between COPD and BYF groups in 32th week.

## Figures and Tables

**Table 1:** TV, PEF, and EF50 in three experimental rat groups ($\bar{x}\pm S$).

Time (weeks)	TV (ml)			PEF (ml/s)			EF50 (ml/s)		
	Control	COPD	BYF	Control	COPD	BYF	Control	COPD	BYF
0	2.83 ± 0.56	2.87 ± 0.47	2.93 ± 0.56	14.62 ± 2.96	14.45 ± 2.98	14.82 ± 3.23	1.72 ± 0.22	1.72 ± 0.25	1.71 ± 0.32
4	2.83 ± 0.44	2.51 ± 0.39	2.54 ± 0.44	14.81 ± 2.88	11.15 ± 2.76	11.43 ± 2.96	1.75 ± 0.24	1.47 ± 0.23	1.44 ± 0.29
8	2.84 ± 0.38	2.04 ± 0.51	2.05 ± 0.43	15.09 ± 2.56	10.02 ± 2.54	10.01 ± 2.48	1.76 ± 0.25	1.04 ± 0.27	1.02 ± 0.26
12	2.87 ± 0.43	1.96 ± 0.41	2.12 ± 0.38	15.40 ± 3.02	9.96 ± 2.83	11.34 ± 2.63	1.84 ± 0.24	0.94 ± 0.28	1.09 ± 0.22
16	2.89 ± 0.44	1.92 ± 0.42	2.16 ± 0.35	15.41 ± 2.75	9.91 ± 2.94	11.93 ± 2.83	1.91 ± 0.27	0.95 ± 0.27	1.16 ± 0.28
20	2.93 ± 0.41	1.90 ± 0.46	2.24 ± 0.46	15.41 ± 2.24	9.95 ± 2.79	12.47 ± 2.77	1.94 ± 0.26	1.01 ± 0.27	1.25 ± 0.28
24	2.96 ± 0.31	1.92 ± 0.24	2.30 ± 0.42	15.60 ± 2.57	10.41 ± 1.52	12.47 ± 1.91	1.95 ± 0.23	1.02 ± 0.24	1.32 ± 0.36
28	2.96 ± 0.33	1.97 ± 0.35	2.27 ± 0.33	16.02 ± 1.72	11.06 ± 2.28	14.44 ± 2.71	1.96 ± 0.28	1.07 ± 0.21	1.39 ± 0.32
32	2.97 ± 0.36	1.99 ± 0.34	2.35 ± 0.36	16.50 ± 1.79	11.05 ± 2.01	14.83 ± 1.66	2.06 ± 0.20	1.08 ± 0.24	1.46 ± 0.34

**Table 2:** Biomarkers selected from DMs from two time points (weeks 20 and 32).

	Metabolite	RT (min)	*m/z *	COPD/control			BYF/COPD		
				VIP	*t-*test	fold	VIP	*t-*test	Fold
Week 20	2-Methoxyestradiol	5.267	303.1911	1.81	0.001	0.54	2.14	0.004	−0.44
	20-Hydroxy-PGE_2_	5.9	369.2265	2.08	0	0.63	2.27	0	−0.41
	5-HEPE	8.91	319.2247	2.08	0.001	0.54	1.73	0.03	−0.3
	7-Oxo-11-dodecenoic acid	6.041	213.1483	1.48	0.03	0.35	1.93	0.01	−0.34
	Acetyl-L-leucine	5.12	174.1117	1.56	0.007	0.5	1.99	0.01	−0.48
	Arachidonic acid	9.633	305.2476	1.51	0.038	0.38	1.96	0.01	−0.38
	Eicosapentaenoic acid ethyl ester	7.161	331.2667	1.71	0.005	0.48	1.93	0.01	−0.34
	Linoleic acid	8.913	281.2394	2.04	0	0.52	2.09	0.01	−0.39
	Lipoxin A5	6.676	351.2163	1.9	0.001	0.48	2.09	0.01	−0.38
	N-Acetyl-L-phenylalanine	5.159	208.0964	1.65	0.017	0.45	1.95	0.01	−0.5
	N-Heptanoyl-homoserine lactone	5.026	214.1431	1.59	0.01	0.46	2.03	0.01	−0.45
	N-Nonanoyl-L-homoserine lactone	5.171	242.1745	1.61	0.018	0.49	1.92	0.01	−0.41
	Phenylpyruvic acid	5.08	165.0595	1.37	0.019	−0.68	1.48	0.03	0.6
	*α*-D-Fucose	5.292	165.0755	1.21	0.024	0.35	1.61	0.03	−0.27

Week 32	Acetylcholine	0.589	146.1169	1.64	0.01	0.43	1.86	0.01	−0.49
	PC (18:1)	11.102	522.3559	1.96	0	−0.25	1.75	0.01	0.38
	Sphinganine	7.917	302.3056	1.57	0.03	−0.42	1.5	0.03	0.52
	Succinic anhydride	0.847	101.0237	1.2	0.04	0.45	1.7	0.01	−0.64
	Arachidonic acid	9.633	305.2476	1.72	0.02	0.49	1.36	0.02	−0.31

**Table 3:** Pathway enrichment analysis using MetPA.

Time	Pathway	Total Cmpd.	Biomarker	*P* value	Impact
Week 20	Biosynthesis of unsaturated fatty acids	42	2	0.016729	0
	Phenylalanine, tyrosine, and tryptophan biosynthesis	4	1	0.019843	0
	Linoleic acid metabolism	5	1	0.024751	1
	Phenylalanine metabolism	9	1	0.044172	0.24074
	Arachidonic acid metabolism	36	1	0.1668	0.32601
	Steroid hormone biosynthesis	70	1	0.30185	0.0068

Week 32	Glycerophospholipid metabolism	30	2	0.002587	0.04444
	Sphingolipid metabolism	21	1	0.058643	0.14286
	Arachidonic acid metabolism	36	1	0.098923	0.32601
	Biosynthesis of unsaturated fatty acids	42	1	0.11467	0
